# An interdisciplinary nationwide complex intervention for lifespan neurodevelopmental service development: Underpinning principles and realist programme theory

**DOI:** 10.3389/fresc.2022.1060596

**Published:** 2023-01-30

**Authors:** Donald Maciver, Marion Rutherford, Lorna Johnston, Eleanor Curnow, Marie Boilson, Mairéad Murray

**Affiliations:** ^1^ School of Health Sciences, Queen Margaret University, Edinburgh, United Kingdom; ^2^ Additional Support for Learning Service, Communities and Families, City of Edinburgh Council, Edinburgh, UK; ^3^ Fife Health and Social Care Partnership, Whytemans Brae Hospital, Kirkcaldy, United Kingdom

**Keywords:** autism, neurodevelopmental, pathway, assessment, diagnosis, ADHD, neurodiversity

## Abstract

**Background:**

People seeking support for neurodevelopmental differences often report waiting too long for assessment and diagnosis, as well as receiving inadequate support in educational and health settings. The National Autism Implementation Team (NAIT) developed a new national improvement programme in Scotland, focusing on assessment, diagnosis, educational inclusion, and professional learning. The NAIT programme was conducted within health and education services across the lifespan for a range of neurodevelopmental differences, including autism, developmental coordination disorder, developmental language disorder, and attention deficit hyperactivity disorder. NAIT included a multidisciplinary team, with the involvement of an expert stakeholder group, clinicians, teachers, and people with lived experience. This study explores how the NAIT programme was planned, delivered, and received over three years.

**Design:**

We performed a retrospective evaluation. We collected data from review of programme documents, consultation with programme leads and consultation with professional stakeholders. A theory-based analysis was completed, drawing on the Medical Research Council Framework for developing and evaluating complex interventions, and realist analysis methods. We developed a programme theory of the contexts (C), mechanisms (M), and outcomes (O) influencing the NAIT programme, based on comparison and synthesis of evidence. A key focus was on identifying the factors that contributed to the successful implementation of NAIT activities across different domains, including practitioner, institutional and macro levels.

**Results:**

On synthesis of the data, we identified the key principles underlying the NAIT programme, the activities and resources utilised by the NAIT team, 16 aspects of context, 13 mechanisms, and 17 outcome areas. Mechanisms and outcomes were grouped at practitioner level, service level and macro level. The programme theory is pertinent to observed practice changes across all stages of referral, diagnosis and support processes within health and education services for neurodivergent children and adults.

**Conclusions:**

This theory-informed evaluation has resulted in a clearer and more replicable programme theory that can be used by others with similar aims. This paper illustrates the value of NAIT, as well as realist and complex interventions methodologies as tools for policymakers, practitioners, and researchers.

## 1. Introduction

Those with neurodevelopmental differences are often at greater risk of unemployment, social exclusion, educational underachievement and poorer mental health (1–5). Attention is required to reduce adverse events, ensure supportive environments, and to meet the calls of people with lived experience for improved professional and societal responses across the lifespan (6).

Whilst there is considerable controversy and debate in this area, approaches to support have progressed from a single-focus approach (e.g., for autism alone) to approaches that include an understanding that neurodevelopmental differences very frequently co-occur and overlap (7). A primary consequence of this new way of thinking is the shift in practice away from a “single condition” focus towards “neurodevelopmental” pathways (8). This has led to changes in multi-disciplinary and multi-agency approaches to referral, assessment, diagnosis, and supports. Within this new way of working, there is a need for change, professional learning and resources to help teams across sectors develop new practices and skills. As services move towards the adoption of a neurodevelopmental approach, this identifies new questions, challenges underlying philosophies, and generates a need for practical solutions and tools.

The National Autism Implementation Team (NAIT) led an initiative that aimed to bridge the evidence and policy to practice gap and facilitate whole systems change in neurodevelopmental approaches in Scotland for autism, developmental coordination disorder (DCD), developmental language disorder (DLD), intellectual disability (ID), fetal alcohol spectrum disorder (FASD) and attention deficit hyperactivity disorder (ADHD). The scope included all statutory health and education services for infants, children, adolescents and adults in Scotland who might require support due to neurodevelopmental differences. The programme was government funded and aimed to facilitate changes in lifespan assessment and diagnosis, educational inclusion, participation and inclusion of neurodivergent people, inter-sectoral and multi-agency working, and use of evidence-informed supports.

Clinicians, educators, and researchers contributed to NAIT activity, with the active involvement of people with lived experience. An interdisciplinary programme was developed, comprising evidence synthesis, working groups, professional learning, publication, practice development, and activism to influence services, practitioners and policymakers.

This paper aims to develop an understanding of NAIT as a complex intervention. An intervention can be complex because of possessing numerous components, multiple targets, and/or involvement of large numbers of groups, settings, or levels (9). It is often difficult to understand how complex interventions work. There may be competing hypotheses about important aspects, outcomes, or processes. Research must therefore extend from “effectiveness” to theorising how and why interventions work (9). A set of key elements should be identified, as insistence on continuing fidelity to multiple, incorrect, or ineffective components can obstruct implementation and innovation (10).

Improving health and education service provision for neurodivergent people, on a national level, is a prime example of a complex intervention. Comprehensive methods to understand such interventions are needed. We followed the Medical Research Council's complex interventions development framework (9). The framework recommends a foundation in “programme theory” (9), an idea from realist evaluation, where programme planners elucidate underpinning principles and processes of programmes (11–14). Programme theories describe how an intervention causes its effects, through which “mechanisms” and under what “contexts” (13). In other words, realist methods identify the “active ingredients” or most important parts of programmes. The MRC framework also strongly stresses partnership (15), emphasising identification of priorities, problems, and solutions with stakeholders (16).

### 1.1. Language statement

We use identity first language wherever possible, as this has been the strong preference of lived experience stakeholders working with the NAIT team. The term “neurodevelopmental disorders/diagnoses/conditions” are medical terms referring to diagnostic practices associated with ICD-10 and/or DSM classifications for diagnostic classification of autism, ADHD, DCD, ID, FASD and DLD. “Neurodevelopmental differences” is a preferred alternative term used to refer to people who may have the above diagnoses but uses language that avoids pejorative labelling and association with words such as “disorders” or “conditions.” “Neurodivergent person/people” is an identity first and non-medical term which has emerged from the wider stakeholder community as an umbrella term for describing people who think differently to those who have “typical” brain functioning and here focusses on autism and the other neurodevelopmental differences (as described above) that are common among those who describe themselves as neurodivergent. In the NAIT programme we focus on lifelong neurodivergence which forms a permanent part of a person's thinking style and way of being. Again, use of this terminology is non-pejorative and supports the idea that people are different, but do not have “disorders”, “conditions” or “illnesses.” For autism, terms such as “autistic person” are preferred to terms such as “person with autism” or “autistic spectrum disorder.” Use of such language confers the view that autistic people do not “suffer from” autism, and do not have a “disease” or “condition.” Being autistic is an integral part of their personhood, and is not an illness that can be removed, “fixed” or “treated.”

## 2. Methods

### 2.1. Aim

The current study examined the efforts of NAIT focused on improving professional practice in education and health services. We aimed to identify the “active ingredients” of NAIT, which represents the theory of change, programme theory, or theory of how outcomes were achieved. A primary focus was to understand how NAIT had been successful in developing, negotiating and continuing to influence health and education practices nationally. It was important to understand what processes and factors were pivotal, as well as identifying and synthesising key success features. Evaluation of direct interventions provided by clinicians or teachers, or outcomes for service users or families, were not the focus of this phase of the research.

Our key question was what aspects of the NAIT implementation, staffing and delivery had been most successful in influencing changes in practice? The analysis therefore aimed to explore and synthesise: (1) the key principles underpinning the NAIT programme, (2) activities, resources and intervention utilised by NAIT, (3) contextual barriers and facilitators, and (4) processes (mechanisms) that had driven outcomes. We focused on three levels: the practitioner, the institutional/organizational level, and wider structures or macro level.

### 2.2. Setting

Scotland is the second largest country in the United Kingdom, with an estimated population >5 million. Scotland has an extensive and comprehensive publically funded education service for children aged 3–18, with a relatively small private provision. Scotland also has an extensive and comprehensive state health system, with limited private alternatives. Most health and education services are provided locally, including schools, hospitals, and other therapeutic, diagnostic and allied health services.

The school system in Scotland aims to be highly inclusive. Policy mandates for universal inclusive practice and is based on a “presumption of mainstream” where children should be educated together wherever possible. Almost all children attend “mainstream” or typical community schools. More complexity of need may trigger external agencies, including health professionals, psychologists, and social workers. Multidisciplinary and multiagency working is mandated by legislation and guided by policy, with numerous processes and statutory instruments to support this. However, there are very substantial local differences in practice, and nationally the health and education systems are almost entirely organisationally separate, with different funding structures, practice locations, staff and training.

For children who may have neurodevelopmental differences, over the age of three, parents often access early assessment and support through the team around the child in the education setting. For children under 3, initial conversations and assessment might be via the health visiting team or community allied health professionals (AHPs). At all ages, they may then be referred for further specialist medical assessment and/or to other highly specialist services, AHPs, teachers, psychologists, charities, advocacy organisations and/or community support organisations. Whilst policy describes coordination of this support, consistent implementation remains a challenge.

For adults, general practitioners (family doctors) act as gate keepers to specialists in mental health and psychiatry. People seeking assessment and support for neurodevelopmental differences often cannot access specialists directly. Long-term follow-up and management mainly occurs in primary care, frequently under "shared care" arrangements with psychiatry, mental health, intellectual disability, and some specialist third sector provision. There can be substantial variations in local practices such as prescribing practices, clinics, and types and availability of specific services. Assessment and support should include consideration of differential diagnoses (including other neurodevelopmental and mental health needs) and a full generic assessment. There should be local practice protocols between mental health teams and primary care.

Various issues exist at a local and national level in child and adult services including long waiting times, difficulties with staff recruitment and staff shortages across a range of professions including teaching, psychiatry, and mental health nursing. There is a move towards developing neurodevelopmental pathways. For children, including neurodevelopmental pathway guidance (8) and Scottish Government national neurodevelopmental specification and health standards of care (17). At the inception of NAIT, some areas of Scotland had implemented neurodevelopmental pathways for children, and a majority were undertaking work to move in this direction, in partnership with education and social care partners, with the aspiration of taking an integrated approach. For adults in Scotland there were no formal adult neurodevelopmental pathways. Intellectual disability and adult mental health services predominated. Co-occurrence of neurodevelopmental differences was somewhat recognised, but adult services were inconsistent in giving routine consideration to co-occurring neurodevelopmental differences. Demand for services (especially in autism and ADHD) often exceeded capacity to meet needs with limited provision outside of adult secondary care. Allied health professionals, specifically occupational therapists and speech and language therapists, were under-represented in these services.

### 2.3. Ethics

This work was carried out in accordance with the relevant ethical standards of institutional and national practice in Scotland and following the Declaration of Helsinki. University or NHS ethical approval was not required for this study as it involved the retrospective collection of service improvement information from professionals and clinicians. No patient identifiable information was collected in this study.

### 2.4. Data collection

Data collection involved document review, consultation with programme leads, and consultation with professional stakeholders who had first-hand knowledge of engaging with NAIT and implementing ideas in practice.

### 2.5. Document review

At the time of writing, NAIT had been operating from 2019 to 2022. Documents covered material across this time period, including agendas, meetings, manuals, website material, infographics, diagrams and reports. Email communications between the programme developers and funders and other stakeholders were also made available. Document review provided candidate ideas for inclusion in later analyses, and a historical overview of NAIT's development.

### 2.6. Consultation with programme leads

Consultation with programme leads (including staff who had left the team) involved clarifying and identifying NAIT components and the relationships between them. This helped to narrow down the contents and identify the “active ingredients” from the perspectives of those responsible for programme leadership.

### 2.7. Consultation with professional stakeholders

Two videoconference 1-hour workshops were conducted focusing on the implementation of NAIT in practice, views on effectiveness of the NAIT programme, and future maintenance. Additional videoconference interviews were completed with autistic professionals, following the same question format, with an additional opportunity to provide email feedback (if desired). Stakeholders were recruited through open invitation, aiming to include individuals who had first-hand knowledge of working with NAIT. In total, forty stakeholders were included. Stakeholders included allied health professionals (occupational therapists and speech and language therapists); education professionals; managers; psychologists; medical staff (paediatricians, psychiatrists, nurses); mental health staff; third sector staff; Scottish Government representatives; and professionals with lived experience of neurodevelopmental differences. Individuals from large urban areas, remote, rural and island communities were included.

### 2.8. Analysis

A theory-based analysis was completed, focusing on NAIT as a complex intervention, using realist analysis methods (9, 14). Realist analysis is an interpretive, theory-driven narrative summary which applies realist philosophy, the main assumptions being that interventions work through mechanisms (M) in different contexts (C) leading to outcomes (O) (13). Interventions work because individuals make decisions in response to the intervention. The reasoning and actions of individuals in response to resources or activities is what “causes” the outcomes and these “mechanisms” are what “causes” change (11, 18, 19). External factors (e.g., policy, environment or cultures) either favour or disfavour activation of mechanisms (this is “context”) (13). Outcomes are any outcome of interest to funders, leaders or stakeholders. Together, the identification of context, mechanisms and outcomes represents the main output of the analysis or “programme theory.”

The analytic procedure is summarised in Table 1. Through analysing documentary and transcribed workshop and interview data, the focus was to identify the mechanisms which were generating outcomes and to demonstrate what aspects of context matter. The procedures focused on coding and thematic analysis, involving familiarisation, identification of a framework, coding according to the framework, and charting, mapping and interpreting (20–22). Coding for “context” focused on eliciting the factors that could favour or disfavour the activation of mechanisms. “Mechanisms” (which represent the theory of change) were identified next. Here, the key analytic question related to “active ingredients” and explanations or justification of why outcomes had been achieved. “Outcomes” were the intermediate outcomes directly linked to mechanisms, and participants' views on broader changes in any area, services or staff (e.g., skills, knowledge, systems, structures, leadership, mind-sets and attitudes) and how these had been influenced by NAIT to improve practice. New concepts were identified and incorporated into the coding framework as required. As the analysis progressed, findings were further classified at macro (e.g., government and policy), institutional (e.g., hospitals and schools) and practitioner levels (e.g., individual teachers or health professionals), as a useful rubric for NAIT's multilevel operations. Final labels were then assigned to each area and the narrative summary was written. Overarching or “guiding principles” were a key feature of the programme as articulated by leaders. We present these in the results, as they comprise an important facet of the NAIT programme theory. Activities and implementation strategies of NAIT were also identified and synthesised.

**Table 1:** Analysis stages.

Stages	Description	
Familiarisation	Full team reads documents and meets to discuss over several sessions.
Framework identification	Full team confirms coding framework, using mechanism, context and outcome and as sensitizing concepts, and macro, institutional and practitioner level as organising concepts
Coding	DM, MR, and EC collated the data into codes
	- **Mechanism:** explanations or justifications leading to outcomes i.e “active ingredients.” - **Context**: aspects that favoured or disfavoured mechanism activation - **Outcome:** outcomes linked to mechanisms	- **Macro:** national, government and policy level - **Institutional:** schools, hospitals and other relevant organisations level - **Practitioner:** individual practitioner level
Charting, mapping & finalisation	DM developed a written account of the analysis and engaged in discussions with the full team – the presentation and analysis was agreed by all authors.

### 2.9. Rigour and trustworthiness

To ensure rigour and trustworthiness interpretations were recorded alongside quotes to maintain an audit trail. The researchers were aware that an “insider” perspective may have influenced the analysis. The researchers used discussion and reflection, and were supervised by an experienced qualitative researcher to identify and record thoughts, feelings, attitudes, and reactions. Regular meetings were held, and interpretations shared across the research team and wider group of collaborators. Refinement of the analysis continued until agreement was reached. There was a point at which no new mechanisms, contexts, or outcomes emerged i.e., saturation was attained.

### 2.10. Patient and public involvement

NAIT was inspired by needs and interests of stakeholders from the neurodevelopmental community, including young people and adults with lived experience, families and user groups. Neurodivergent people have been involved in a range of ways, including as researchers in the NAIT team. Overall, we aimed for meaningful coproduction, and the highest level of community participation that was realistic and achievable within the programme (23).

## 3. Results

First, the NAIT underlying principles are presented (Table 2), followed by NAIT activities and resources, including content developed by NAIT (Table 3) and pre-existing interventions used (Table 4). Next, the realist analysis is presented, covering the “programme theory” of mechanisms and contexts influencing outcomes (see Table 5 for an overview, and Supplementary Additional File S1 for a figure.).

**Table 2:** NAIT principles.

Principle	Description
Diagnosis Matters	No support should be diagnosis dependent, but feedback from neurodivergent people indicates it is important. Delayed, missed or misdiagnosis impacts quality of life, mental health and support. Diagnosis provides self-understanding, identity, peers, access to information and can enhance an awareness of supports.
Environment First	The fundamental basis of all effective support is having the right expectations and adjustments in the physical and social environment in everyday settings.
Language & Mindsets Matter	Negative experiences can be reduced through changes in language and mindset. A key message is ‘we were expecting you.’ We should expect to meet neurodivergent people in everyday life. Professionals should expect to meet neurodivergent people and should anticipate their needs.
Neurodevelopmental Lens	Neurodevelopmental differences frequently co-occur. Employing a neurodevelopmental lens means taking account of sensory and communication preferences and individual thinking styles. We can approach everyone with an inclusive mindset and the right approach. We invest in developing neurodevelopmental services strategically aligned across the lifespan. National neurodevelopmental pathways are required to frame and shift practice.
Nothing about us without us (co-production & partnership)	Neurodivergent people are included throughout. There is a need for co-production and involvement at all stages. For children and people with limited communication, families are also advocates and partners.

**Table 3:** NAIT activities and resources 2019–2022.

Activities & resources	Description
Multidisciplinary team	Representing 5–6 whole time equivalent staff, the NAIT team included expertise in speech and language therapy, education, psychiatry, occupational therapy, autism, health systems research, complex interventions development, evaluation methodologies and researchers with lived experience.
‘Divergent partners’ reference group	Meetings with a user group of adults with lived experience for consultation, review and to inform the development of the NAIT programme.
Meeting & reporting to government ministers	Regular meetings with senior government officials to present progress, receive feedback and discuss plans.
NAIT resources and practice guides	Topic guides, including evidence (where possible/relevant) for practitioners with practical information and strategies (e.g., remote assessment, hyperacusis, anxiety related absence from school).
Professional learning activities and training	A rolling series of multi-agency events on autism and other neurodevelopmental differences. Learning was at three levels graded from introductory-expert and provided by a multi-disciplinary team. Tailored content, based on local priorities, was available on request.
Professional support activities	Individual meetings with the NAIT team were available on request (e.g., local area leads from health or education) including support, problem solving, feedback and question and answer sessions.
Covid-19 guidance	A range of published material for staff, service users and families (e.g., stay at home advice for parents, return to school, and furloughed workers).
Initial teacher education (ITE) framework	An evidence-informed framework for initial teacher education on autism and neurodevelopmental differences was collaboratively developed by NAIT and a range of stakeholders, and will be used by all ITE institutions (universities) in Scotland.
Children & young people's evidence based practice guide	A guide to support selection of developmentally appropriate, evidence based approaches and supports. The guide contains a map of common evidence based autism interventions, organised by developmental level and target areas.
Children's neurodevelopmental pathway framework	A pathway including evidence based recommendations in relation to clinical neurodevelopmental assessment, diagnosis and interventions. The pathway includes referral management, triage, screening and surveillance, early development and history taking, contextual assessment, direct observation, and mapping to diagnostic criteria.
Adult neurodevelopmental assessment workbook	A guide to assist assessment of adults whose presentation is suggestive of a neurodevelopmental difference, with a particular focus on ADHD and autism. It includes risk assessment, evidence based tools for assessment, neurodevelopmental history, medical assessment including prescribing, and template letters supporting workplace adjustments.

**Table 4:** Pre-existing interventions utilised by NAIT.

Activities and resources	Descriptions
CIRCLE Framework (universal inclusive practice)^a^	A manualised, evidence-informed resource, for teachers, to support universal inclusive practice in schools. A ‘train the trainer’ set of materials and videos was developed by NAIT to facilitate implementation. Badged online professional learning modules were also developed with a government agency and made available to all Scottish teachers.
SCERTS (specialist assessment and planning framework)^b^	A manualised evidence-informed and multidisciplinary framework for specialist assessment and planning. It includes supports at three developmental stages from pre-verbal to conversational. Three three-day training sessions were commissioned by NAIT and delivered by SCERTS authors. Comprehensive online documentation was also developed by NAIT to facilitate implementation tailored to the Scottish context.
Parent Support Intervention^c^	An evidence-informed framework to reduce parent stress and improve quality of life. Content includes a group structure with information sessions, post diagnostic support and home based support. Brief online documentation was developed by NAIT to facilitate implementation.
Visual Supports Resource Intervention^d^	A manualised evidence-informed set of resources providing a core, consistent visual support symbol set. Comprehensive online documentation was developed by NAIT to facilitate assessment and implementation.

^a^ (24, 25).

^b^ (26, 27).

^c^ (28).

^d^ (29).

**Table 5:** Realist matrix for the NAIT programme (programme theory).

Context^a^	Mechanisms^b^	Outcomes^c^
Policy & directives Attitudes & mind-set Early career professional development needs Knowledge gaps & outdated perspectives Information overload Unsure which sources to ‘trust’ Current levels of multi-disciplinary working (particularly health & education) Professional silos For children & young people: diagnostic services already in place, some pathways For adults: minimal diagnostic services in place, minimal pathways Different funding models nationally Staff recruitment & retention Perception that change is difficult Lack of time & ‘too busy’ Fear of the unknown COVID 19 pandemic recovery	**Macro Mechanisms** - Engage routinely with a range of national strategic groups, leadership groups, & cross sector groups - Engage the apparatus of government, work with senior government officials, & respond to their requests	**Macro Outcomes** - Improved responsivity to current issues & needs - Programme team perceived as leaders - Influence on policy & national practice - Improved links & engagement with policy & government - Allocation of funding
	**Practitioner Mechanisms** - Be action orientated - Be a persuasive & trustworthy source of information across practice & research - Provide accessible, concise, engaging, evidence-informed information, with consistent key messages - Be an accessible team - Challenge & support - Develop debate & consensus on community acceptable cross sector language & terminology - Model expected behaviours	**Practitioner Outcomes** - Increased responsibility, motivation, empowerment & performance of staff - Improved likelihood of strong & sustainable engagement with key NAIT ideas across staff & practice communities - Agreed language that is acceptable to stakeholders - Inclusive mindset, inclusive staff - Informed & educated staff - Development of leaders
	**Institutional Mechanisms** - Have a national focus - Promote universal inclusive practice - Provide a systematic framework to embed sustainable practices through high quality professional learning materials - Facilitate & lead multi-professional networks with locally owned change programmes	**Institutional Outcomes** - Consistent use of recognized evidence based strategies & approaches - Changes in relationships within & across organisations - Increase in multi-agency/multi-disciplinary approach in planning, development & implementation - Robust local & national neurodevelopment networks - Introduction of policies, changes in social norms, &/or normalisation of NAIT recommended practices - Improved quality & consistency of support delivered for children & adults

^a^ Context: factors which favour or disfavour the activation of mechanisms.

^b^ Mechanisms: processes underpinning changes in outcomes – ‘active ingredients’ which when activated cause changes in the thinking, behaviours and reasoning of actors, in this case professional staff working in the health and education sector.

^c^ The direct role of NAIT is to provide support to enable practitioners across sectors to develop practice in this field. Outcomes are focused on practice changes associated with NAIT, and to understand the impacts of NAIT in this ‘audience’ which is the professional staff working in this field. Evaluation of direct interventions provided by clinicians or teachers, or outcomes for service users or families, were not the focus of this phase of the research.

### 3.1. NAIT principles

NAIT principles (Table 2) are features around which the NAIT programme was developed according to programme leads. Principles were used to guide development and communicate intentions and values.

### 3.2. NAIT activities and resources

Tables 3, 4 describe NAIT activities and resources, including content developed by NAIT (Table 3) and pre-existing interventions used (Table 4). All materials were online (30), as according to programme leads, resources developed with public funds should be freely available. Multiple communication methods were used, including events, handbooks, “quick read” documents, social media, email, newsletters, publications, and research summaries. Regular consultation meetings were held with people from relevant communities, and with the government.

### 3.3. Realist analysis

The next phase of the analysis was to produce the realist analysis. Based on the evidence, we constructed a programme theory that depicts mechanisms and contexts influencing practice outcomes (Table 5 for an overview, and Supplementary Additional File S1 for a figure). The analysis represents the theory of how the NAIT programme produced its change(s). Contexts are described first, followed by mechanisms and outcomes. Indicative quotes capturing the views of included stakeholders are in Supplementary Additional File S2.

#### 3.3.1. Context

Context represents the factors which favoured or disfavoured the mechanism activation. Context describes the environment in which the programme operates and external factors that interact with it and influence it.

#### 3.3.2. Macro context

Context at the macro level was supportive. There was a climate of significant debate around neurodiversity. Basic rights, such as inclusion in school, or opportunities to work, not being met, and the effect on individuals, was understood. The policy context was strong and recognised as such by stakeholders. There were government policies and directives focussed on improving outcomes. A policy vision describing enablement, empowerment, and self-actualisation, with levers such as self-directed support, with strong user group representation, was apparent.

#### 3.3.3. Practitioner and institutional context

There were supportive practice values around wanting to do better (e.g., more “joined up” working), and a commitment to improve outcomes. Locally, there were examples of supportive policy and practice. Aspects currently working well described by stakeholders included early communication support, autism outreach, some reducing waiting lists for diagnosis, third sector collaboration, peer support, parent support, and examples of joint working. Some aspects of inclusive practice in education were working well, e.g., some multidisciplinary planning processes.

There was however a disconnection between policy ambition and reality. Joint pathways between health and education were commonly viewed as beneficial, but widespread inconsistency meant that levels of joint working were variable. Consistent use of evidence-informed interventions was also lacking. Practitioners stated they struggled to know which sources to trust and which “evidence based” interventions were effective or appropriate. Some practices were focused on participation, inclusion or had a primary focus on environment. However, other interventions were embedded in a climate of negative representations of neurodivergent people. Such responses would be focused on changing individuals, trying to “fix” them or encourage neurotypical behaviour. Interventions to allow people to access community activities, manage transitions, and support to (re)enter work were less routine.

The diagnosis and pre-diagnosis phases were understood to be of fundamental importance, and a “neurodevelopmental” pathway for diagnosis was seen as desirable by many. However, there were few formal pathways for adults with ADHD or neurodevelopmental differences who did not have significant co-occurring mental illness, with long waiting lists. There were neurodevelopmental pathways for children, but consistency was lacking.

The attitudes of some education staff (e.g., teachers who may not think neurodivergent children should be in mainstream education) and adult mental health staff (who may not have a framework to understand neurodevelopmental differences or view this as core practice) were considered by stakeholders to be particularly difficult contextual challenges. A final aspect related to adult service demographics, where ADHD was pervasive, and perceived to be overwhelming service capacity. This was compounded by a lack of skills and interventions in adult services.

Common change factors, such as perceptions that change is difficult, fear of the unknown, lack of time, funding and staff turnover were also identified as aspects of context.

#### 3.3.4. Mechanisms

The identified mechanisms at macro, institutional and practitioner levels are described. These represent the “active ingredients” of the NAIT programme, which, in combination with the activities and resources, and key principles as described, were responsible for driving change. A summary, along with identified outcomes, is in Table 3.

##### 3.3.4.1. Macro level mechanisms

The realist matrix at the macro level included two mechanisms. Macro mechanisms capture the system of government and policy which NAIT was part of. These mechanisms were related to NAIT's status as a government funded and endorsed programme, as well as liaison and communication with government and other national bodies.

###### 3.3.4.1.1. Macro mechanism 1: “Engage routinely with a range of national strategic groups, leadership groups, and cross-sector groups”

This mechanism related to NAIT providing strategic input and leadership in a range of forums, leadership groups and cross sector activities. The NAIT team was facilitating multi-disciplinary networks at a senior level and providing input to strategy and development. Through these processes NAIT staff acquired knowledge of current priorities, which helped them to understand how to proceed. The programme staff were also perceived to be challenging and critiquing multi-disciplinary practice at a senior/national level. As a result, they became perceived as leaders and, thus, were more likely to influence future practice.

###### 3.3.4.1.2. Macro mechanism 2: “Engage the apparatus of government, work with senior government officials, and respond to their requests”

This mechanism related to the process of creating shared understandings across government departments and government organisations to coordinate efforts to facilitate change, as well as ensuring government commitment to resource provision (e.g., salaries). Directly responding to senior officials, and regularly working with them to identify priorities, supported identification of issues, senior leadership, and government support for change. This mechanism also captured the sharing of specialist knowledge from NAIT to government officials (e.g., responding to requests from government for statistics, reviews of literature or commentary) and government bodies to educate and influence.

##### 3.3.4.2. Practitioner level mechanisms

The realist matrix at the practitioner level included seven mechanisms. These represent the processes which when activated supported outcomes at the practitioner level, for health professionals, teachers and other relevant staff.

###### 3.3.4.2.1. Practitioner mechanism 1: “Be a persuasive and trustworthy source of information across practice and research”

Analysis indicated that there was a perception from stakeholders that NAIT was a credible and trustworthy information source. NAIT included individuals from health and education backgrounds with practical, educational and clinical experience, who were recognisable leaders with specialist knowledge. NAIT also included expertise in research. This combined expertise from different fields led to greater face validity, enabling confidence and greater likelihood of application of ideas and recommendations in practice.

###### 3.3.4.2.2. Practitioner mechanism 2: “Be action orientated”

NAIT was perceived by stakeholders and programme leaders to encourage practical action to advance rapid (where appropriate) changes. The combination of a programme which was perceived to be persuasive and trustworthy, and perceived to be highly focused on action, motivated individuals to make changes in their own practice. Considering context, it was necessary to acknowledge and take into account people's feelings and perspectives when change was difficult, but still encourage change.

###### 3.3.4.2.3. Practitioner mechanism 3: “Provide accessible, concise, engaging, evidence-informed information, with consistent key messages”

According to stakeholders, NAIT materials were more likely to be used in practice because they were perceived to be succinct, thoughtfully presented and (where possible) evidence-informed. NAIT communications were orientated towards key messages and had a strong visual component. The presentation style of materials was noted by stakeholders to be distinctive, engaging and helpful. Consistent key messages (for example guiding principles) supported people's autonomy and offered a meaningful and straightforward rationale for action.

###### 3.3.4.2.4. Practitioner mechanism 4: “Be an accessible team”

In discussion with programme leaders, the NAIT team had explicitly determined to be responsive and held high standards in promptly answering communications, providing individual meetings on request, and onsite visits if appropriate. Stakeholders positively described the team's accessibility and availability of opportunities to reflect on practice. The team's accessibility supported the formation and maintenance of strong working relationships with practice communities, organisations and individuals.

###### 3.3.4.2.5. Practitioner mechanism 5: “Challenge and support”

This mechanism captured the idea that “challenge’ was necessary in addition to support. The “challenge” role NAIT was able to provide was viewed as valuable by stakeholders, encouraging critical reflection, action, change and new solutions. Stakeholders and programme leaders described the team as possessing the necessary expertise, knowledge, attitudes and mind-set to disagree, debate and problem solve with professionals, which supported tackling difficult issues.

###### 3.3.4.2.6. Practitioner mechanism 6: “Develop debate and consensus on community acceptable cross sector language and terminology”

This mechanism captured the ongoing process of change necessary around ideas that language underpins mind-set, attitudes and behaviours, and that outdated or perceived to be offensive language was less acceptable e.g., “autistic people” was preferred to “people with autism”. Consistently using inclusive language was seen to support transfer of values from theory to practice, developing an alignment of people and services across desirable values and ideas. This shift in thinking was challenging in some domains, particularly linked to medical diagnostic practices. Although NAIT materials referenced diagnostic criteria, which are implicitly deficit and impairment focussed and refer to “functional impairment,” the team were committed to finding ways to marry this situation with changing mind-sets, and with the adoption of a neurodiversity paradigm and strengths based approaches.

###### 3.3.4.2.7. Practitioner mechanism 7: “Model expected behaviours”

Expected team behaviours included being considerate, action taking, being evidence-informed, and using inclusive language. This was closely tied to debating and confirming a contemporary and critical national vision. Stakeholders described seeing desired values enacted, with NAIT perceived to be facilitating the transfer of values from theory to practice. Alignment across a range of values and ideas was seen as supporting change. The NAIT programme was perceived to be aligned with what individuals and organisations were trying to achieve, and therefore the team was able to provide leadership.

##### 3.3.4.3. Institutional level mechanisms

The realist matrix at the institutional level included four mechanisms. These represent the processes which when activated supported outcomes at the institutional or organisational level, for example in schools, hospitals or other support settings.

###### 3.3.4.3.1. Institutional mechanism 1: “Have a national focus”

As NAIT is a national multi-professional team, stakeholders found NAIT to be in a strong position to work across agencies, introduce new practices and reduce duplication. This mechanism was strongly supported by setting up and sharing national events and following up with regional events. NAIT's focus on national practice enabled the engagement of organisations in strategic work and implementation of change. This mechanism led to cross-regional involvement, and national connections that supported and led to collaborative working. The credibility and perceived quality of the NAIT team and materials was also seen as beneficial for change at a national level.

###### 3.3.4.3.2. Institutional mechanism 2: “Promote universal inclusive practice”

NAIT leaders described this mechanism in terms of the promotion of a “baseline” universal inclusive practice that should be in place, particularly in education. Consistent use of recognised evidence-informed strategies and approaches (e.g., CIRCLE and SCERTS) was promoted across regions with tailored professional learning opportunities provided to students, practitioners and advanced practitioners. Also important was promotion of a consistent understanding of adjustments for neurodivergent people (e.g., visuals; safe spaces; predictability; anticipatory support and sensory needs), and promotion of the idea that neurodivergent people will be in every setting. This also facilitated promotion of a “social” model of disability, and an understanding of unhelpful physical environments or social structures (e.g., busy corridors, lunch halls, or unstructured times).

###### 3.3.4.3.3. Institutional mechanism 3: “Facilitate and lead multi-professional neurodevelopmental networks”

This mechanism related to facilitating iterative regional/national support events and regional/national multidisciplinary networks. The NAIT team took the lead on preparation, planning, identification of issues, synthesis of views, and partnership to develop solutions/resources. Developing a cycle of national networks followed by regional networks meant local teams were supported to progress with relevant solutions and to have a sense of the “bigger picture.” Networks facilitated a collegial environment between staff. Stakeholders found particularly valuable networks focused on universal inclusive practice and advanced autism practice. Networks were instrumental in supporting locally owned change programmes and supporting naturalistic development of leaders.

###### 3.3.4.3.4. Institutional mechanism 4: “Provide a systematic framework to embed sustainable practices through high quality professional learning materials”

This mechanism was related to the development and publication of “best practice” professional learning materials, as well as the development of consistent key messages. As far as possible, information was also evidence-informed. This included neurodevelopmental service standards/guidelines and new models of working, national professional learning resources for CIRCLE and SCERTS, a national professional learning resource for diagnosis, and national resources for visual supports. An aim was to develop practices which would be sustained over time, so materials were manualised wherever possible and designed to have straightforward application in naturally occurring situations (e.g., classrooms, workplaces, or homes).

## 4. Discussion

In this paper, we describe a complex intervention, implemented over three years. We have shown through describing the programme's underlying principles, and with identification of mechanisms in context, how a relatively small team has been able to deliver a significant programme of work which is having impacts on routine service delivery nationally. The evaluation has also demonstrated the feasibility and sustainability of NAIT ideas and recommended practices. The NAIT programme was underpinned by key principles which encompass the importance of diagnosis, environment focused change, partnership, language and mind-set, rejection of deficit approaches, and a focus on all neurodivergent people.

While the data presented, and programme described herein, supports evidence for effectiveness, care should be highlighted to consider the context of where the program was implemented. The programme is grounded in this context, and overgeneralisation of findings should be avoided. However, our analysis does support transferability to other situations with similar characteristics or issues through its programme theory. Theory driven research on complex interventions leads to refinement of theory across contexts (9). The focus is not the “programme” but the “programme theory,” explicitly operating under the idea that “NAIT” (as applied in the current study, in Scotland) is not reproducible, but the underlying programme theory may be. The NAIT approach would significantly adapt and transform if applied in different contexts, but the key underlying principles, mechanisms and potential outcomes remain pertinent to contemporary neurodevelopmental practices. Thinking and practice continues to move towards neurodevelopmental rather than single focus pathways. A key question is how this can best be achieved when many services are overwhelmed with existing responsibilities and approaches, indicating that new ways of working are desired and necessary.

Only a few studies in the autism or neurodevelopmental field have applied complex interventions or realist-style methods to understand the challenges in this area. Analogous research (31), based on realist literature review methods, and based in the UK, has identified similar findings on key aspects of autism support in health. These key areas were (1) initial recognition; (2) referral and triaging; (3) diagnostic modelling; (4) providing feedback to parents; (5) working in partnership with families; (6) interagency working; (6) and training, service evaluation and development (31). This work, and other recent work (32) also strongly recommends a broader neurodevelopmental approach as being necessary to support efficient service efficiency and positive experiences for people and families. The NAIT programme of work meets several of the calls made in previous realist work (31) for further investigation including research on training and support materials for non-specialist staff, the impact of autism training packages to upskill clinical staff, and wider approaches to integrating services dealing with autism.

A noteworthy factor of the NAIT programme was the good fit of key components with contemporary constructions of autism and neurodiversity. An advanced approach to neurodevelopmental support requires knowledge that neurodiversity encompasses differences in society, not deficits (33), and an engagement with debates around the (in)appropriateness of behavioural and “medical model” interventions. Historically, some interventions may try to “fix” people (e.g., reducing stimming, or increasing eye contact) and there is now understanding of the stress and detriment such interventions can create (34, 35). Alternatively, a focus on participation, environment and real world support is desired by neurodivergent people (6). Facilitation of environmental and participation focused supports were therefore high priority for NAIT, with a focus not on changing the person but finding the right match between individuals and environments. Engagement and promotion by NAIT of such ideas has proved to be attractive to practice communities and people with lived experience, and a key factor in bringing people into the programme and facilitating change.

Research on “live” programmes attempting to make evidence-informed changes in practice is important because there is usually a lag between the development of new knowledge and the implementation of new practice (36, 37). Applied research as opposed to basic research, and research that aims to improve the lives of people within the community is also strongly desired by neurodivergent stakeholders (6). New knowledge, particularly the neurodiversity paradigm, is current, but there are still many gaps between ideas espoused and practices. Successful implementation of neurodiversity-relevant work in real-world settings and the understanding in “how to” enact strategies to successfully translate ideas and reconcile with existing practice is a significant gap (38). Practice-based evidence is needed to address this, rather than tightly conducted empirical research which can serve to widen the gap by perpetuating overly academic or narrow perspectives (39). Our work has identified principles, activities and underpinning mechanisms which can be capitalised on to bridge the theory to practice gap and apply contemporary neurodiversity ideas in practice.

A national focus, with development of networks, and collaboration with pre-existing bodies, were key. National and regional networks aimed to foster locally owned change programmes in line with key values and ideas. A further important step was establishing sustainable delivery. This was supported through ensuring that materials were available online freely. Collaboration with national bodies also provided a vehicle for ongoing integration into routine practice, and longstanding bodies had potential to oversee implementation of NAIT ideas long term. Key activities included autism initial teacher education content which is planned to be implemented by all initial teacher education institutions in Scotland, and “train-the-trainer” material for universal inclusive practice, which aims to ensure that all staff have relevant knowledge.

Altering the health, social care and education “environment” is important. Changes to the work of professional staff is supportive of the health and well-being of neurodivergent people, through facilitating improved quality and consistency of support and reducing health inequalities (40, 41). The types of impacts being described in this study, which include staff awareness, staff knowledge and skills, development of leaders, consistent use of evidence based strategies, and increase in multi-agency or multi-disciplinary working, makes person centred, contemporary, collaborative support more likely. If such practice is being used across Scotland, this means there is increased consistency and quality of provision, as well as consistency in ways of working and ways of thinking that neurodivergent people report to be valuable (6), including practical support, inclusive language, environment focussed adaptation, and strengths based neurodiversity-affirmative assessment.

In order to address the realist methodology concept of “what works well, for whom, under what circumstances” it is helpful to explore the barriers and obstacles to success which were encountered in developing practices, alongside the positives. Some of the most difficult issues to untangle were related to language and mind-set. Bringing together deficit focussed diagnostic criteria with a strengths focussed neurodiversity-affirmative assessment paradigm is very difficult. Clinicians are familiar and comfortable with deficit focussed tools, which are strongly recommended by single-focus clinical guidelines (42). Many tools designed to take a broader neurodevelopmental approach also use deficit focussed language of diagnostic criteria (43). As far as we are aware, there are no self-report tools for young people or adults that use neurodiversity-affirming or explicitly non-pejorative language. This remains an area in need of development, and a continual challenge, especially in health services.

A further key complexity is why diagnosis is needed if it perpetuates a deficit focussed approach. Our rationale is that there is a clear indication from neurodivergent people and the literature that diagnosis is important (44–46). Diagnostic labels are a shorthand for understanding the types of adaptations and supports that might be required, and provide access to self-understanding and a community of peers. Diagnosis can be stigmatising, but rather than being an inherent property, this reflects stigmatising attitudes in society, and a lack of acceptance and understanding of diversity.

A further area of significant complexity was the adult service context in Scotland. In line with UK-wide trends (47), demand for provision was extremely high, particularly for ADHD assessment and supports. Practitioners leading change in adult neurodevelopmental pathways lacked access to evidence-informed approaches, professional learning, and relevant resources for a broader neurodevelopmental approach within more typical “mental health” provision. Environmental modifications are recommended as a first line of intervention for autism and other neurodevelopmental differences across the lifespan (42, 48) and are well understood in some professional groups. However, the full application of these ideas, i.e., that attitudes, understanding, expectations and everyday environments fundamentally and profoundly impact on neurodivergent people, reflects a conceptualisation of neurodivergence that can present challenges to clinicians who may be situated in an individual deficit model (49). This is especially true in adult mental health services where the need for education on autism, ADHD and wider neurodevelopmental approaches is particularly pressing (43, 47).

### 4.1. Future research

Complex interventions research requires a pluralistic evidence base (9), so multiple methods are required. Further realist analysis would provide more theory building. The direct role of NAIT is to support practice change. One can assume an indirect impact on neurodivergent people but multiple variables limit the possibility of evaluation of the extent and nature of this impact currently. Qualitative methods with neurodivergent people would provide useful descriptions. Measures captured on services, particularly fidelity to NAIT principles and uptake of specific recommendations, would also provide useful insights. Key longer term outcomes for evaluation are development of whole system multidisciplinary inclusive services, reduced waits for assessment and supports, competent, confident staff, and most importantly improved lived experience outcomes.

## 5. Conclusions

This evaluation identified the important aspects of a new programme (NAIT) to improve health and education provision for neurodivergent people across the lifespan in Scotland. NAIT was effective in supporting implementation of new working practices and partnership approaches, and was considered acceptable, relevant and feasible by professional stakeholders. As a complex intervention, with many component parts, it was difficult to identify the most important aspects. We explored NAIT with workshops, interviews and analysis of documentation. It would be inappropriate to assume that the findings here apply to all neurodevelopmental supports in all contexts, as there are many different models used internationally. However, the underlying programme theory and key ideas have wider applicability. Analogous activities and resources could be targeted to new contexts, based on the needs of that context. The identified mechanisms represent a multidisciplinary, national approach to facilitating change. Alongside the NAIT principles, this captures a contemporary conceptualisation of neurodevelopmental support, which may be of use to the wider community and could form the core of the theory model for new applications.

## Data Availability

The original contributions presented in the study are included in the article/**Supplementary Material**, further inquiries can be directed to the corresponding author.
