# Supplementary material for: An interdisciplinary nationwide complex intervention for lifespan neurodevelopmental service development: Underpinning principles and realist programme theory
Source: Front Rehabil Sci. 2023 Jan 30;3:1060596. doi: 10.3389/fresc.2022.1060596 (PMC9922833; doi:10.3389/fresc.2022.1060596)
Supplement: Supplementary file 1 [file Table1.pdf]

An interdisciplinary nationwide complex intervention for lifespan neurodevelopmental service development: underpinning principles and realist programme theory

Additional file 2

Contents

Indicative quotes..2

Indicative quotes

Verbatim quotes linked to each area are presented in the table below

**Mechanism**	***Indicative quotes***
**'Engage routinely with a range of national strategic groups, leadership groups, and cross sector groups'**	*"NAIT has a key role in pulling together pieces of work, being a central conduit across agencies, avoiding duplication, due to their credibility with a wide and varied multi-agency and cross agency groups." [Scottish Government Representative]*<br><br>*"They [NAIT] get the mix right between listening, and also not just incorporating everything that everyone says and just ending up with a massive mix…they're quite competent and skilled in terms of managing the political side of things" [Autistic professional]*<br><br>*"[discussing national committee] – the 'critical friend' approach to the [committee] has been really refreshing – broad and flexible and scoped Morgan Review recommendations. Added value to where thinking of [linked national committee]." [Education Manager]*
**'Engage the apparatus of Government, work with senior government officials, and respond to their requests'**	*"Knew [about NAIT] because of work on [Government Strategy]. NAIT is one of the most tangibly strategic things to come out of the strategy – a good outcome." [Third Sector Manager]*<br><br>*"Concur, established strong brand, respected, skills and competencies recognised, NAIT bring something quite unique but you're not seen as a standalone, could be further strengthened by increased links with [committee, in other government department]." [Scottish Government Representative]*
**'Be a persuasive and trustworthy source of information across practice and research'**	*"It's combined expertise from different fields with breadth of experience on diagnostic process, working in schools, has autism lens on everything they do, turning peoples thinking about the way we do things." [Deputy Head Teacher]*<br><br>*"Exceptional quality of resources, very distinctive style and tone, everything is high standard." [Third Sector Manager]*<br><br>*"My impression is that the kind of work is very professional, it's very collaborative. It's done across networks and disciplines which I think is very important and unique in this area" [Autistic professional]*

**'Be action orientated'**	*"Having campaigned around school exclusion it's really positive to have NAIT come in on the back of that - after actions and implementations are agreed. Introducing autism content in ITE [initial teacher education] they were a real driving force around that. Not just practice but tools for strategic change – quickly and in the right way – really invaluable." [Scottish Government Representative]*<br><br>*"NAIT have supported us, recent documentation with the implementation tool - that's what we were going to have to create ourselves, really useful, actually do-able (only 4 pages), manageable and feasible – has moved things on really quickly, really accessible and not just words for the sake of it. Created by people who knew what they are doing and talking about. Anything NAIT comes up with is really accessible and relevant." [Allied Health Professional]*<br><br>*"In the pandemic – NAIT were really proactive and supportive – trying to reduce anxiety – huge positive – definitely added value." [Scottish Government Representative]*
**'Provide accessible, concise, engaging, evidence-informed information, with consistent key messages'**	*"Very high quality, much needed – evidence based, thoughtful and collaboration that goes into it." [Third Sector staff]*<br><br>*"Good quality, really well presented, great for signposting, ideal for signposting education partners, attaching the references a lot on our website." [AHP Service Lead]*<br><br>*"NAIT has become a first port of call for me if I'm looking for something that's got an evidence base, good for all parts of my role – supporting parents – good in lockdown, training, service development, multiagency, strategic planning for Neurodevelopmental pathway – we signpost to a lot of it on our website – keep it coming." [Educational Psychologist]*<br><br>*"Coming from [area] so knew about NAIT from its inception…it's been really good to see that journey, they do great work, great platform to share Evidence Based Practice, multi-disciplinary, people in our service benefitted from the resources." [Depute Head Teacher]*
**'Be an accessible team'**	*"I've luckily had [NAIT staff] come up and co-facilitate a development day with key stakeholders, used NAIT resources for basis of service, resources were invaluable to get things up and running." [Lead AHP, Autism Diagnosis]*<br><br>*"Always incredibly collaborative to work with." [Education officer]*
**'Challenge and support'**	*"I get a lot of support from having conversations with NAIT, I like the challenging of my thinking, sometimes it stops me dead in my tracks, that's a good thing, in your own workplace you can get stuck in a way of being, being challenged in your assumptions is a good thing, time to think stuff through, NAIT are open and willing." [Educational Psychologist]*

	"Useful to pick up the phone and chat to NAIT for advice and support, keeping up to date with pathways, access to skilled professional people." [AHP, Autism Diagnosis]
**'Develop debate and consensus on community acceptable cross sector language and terminology'**	*"Language and terminology, autism, ND getting in a right mess, needs leadership and discussion, language is vitally important risk that some of the, real challenges will get diluted in the malaise of language. Real need for leadership around that." [Scottish Government Representative]*<br>*"Very much so, all the points highlighted in teaching resources, thinking applied to ND pathways, Autism Toolbox works - all align with our view of not taking a 'fix it' approach, reflexive introspective approach. Very aligned. I'd love to get on a t-shirt good things should just happen." [Scottish Government representative]*
**'Model expected behaviours'**	*"Practice of bringing the autistic voice, NAIT is modelling that quite well, something that needs to get better." [Allied Health Professional]*<br><br>*"Developing resources for [my service]– will be referring to materials as really up to date. Style/ language suit what she is trying to convey." [Allied Health Professional]*<br><br>*"Child-centred, holistic, inclusive approach important in Scottish Education, basis of inclusive practice, definitely aligned." [Education Scotland Representative]*
**'Have a national focus'**	*"Where NAIT resources have been absolutely fantastic is when local areas have struggled amongst themselves to align their vision, having NAIT resources as gold standard has helped get on track with what national vision is." [Allied Health Professional]*<br><br>*"NAIT has become a go-to resource, knowledgeable people we can come to with questions, a great national resource." [Clinical Lead AHP]*
**'Promote universal inclusive practice'**	*"Introducing autism content in Initial Teacher Education (ITE) they were a real driving force around that. Not just practice but tools for strategic change – quickly and in the right way – really invaluable." [Allied Health Professional]*<br><br>*"CIRCLE and SCERTS - NAIT have supported us, recent documentation with the implementation tool - that's what we were going to have to create ourselves, really useful, actually do-able (only 4 pages), manageable and feasible – has moved things on really quickly, really accessible and not just words for the sake of it. Created by people who knew what they are doing and talking about. Anything NAIT comes up with is really accessible and relevant." [Allied Health Professional*

	"Added value, SCERTS, and a distinctly different approach and a different framework which they couldn't have found anywhere else. It has added value and supported accreditation with National Autistic Society in schools in [our local authority]." [Education Manager]
**'Facilitate and lead multi-professional neurodevelopmental networks'**	*"One thing we found really helpful, regional networks not just for SCERTS frameworks but also local practitioners and what experience NAIT bring, led to us making informed choices. You provided things to back these up. NAIT support and resources were helpful as they had a national view, little practical bits , don't buy CD, buy manuals, seemed quite small but helpful and saved us a bunch of hassle, richer than that, partnership. NAIT and local helps us co-identify key improvement areas, helped steer, helped us make that leap of faith." [Educational Psychologist]*<br><br>*"NAIT resources are really helpful for joined up approaches with educational partners, especially during COVID/lockdown where training was restricted, has really helped us to move forward to roll out CIRCLE and SCERTS and work closely with educational partners. Really positive for us here." [Allied Health Professional]*
**'Provide a systematic framework to embed sustainable practices through high quality professional learning materials'**	*"Accessible, high standard publications, resources are used often, they distil complex concepts into very clear language. Frequently , at least once a week, used with families and educational establishments, very much a go-to for our service…absolutely superb." [Educational Psychologist]*<br><br>*"Excited about the training framework, having a really cohesive programme for rolling out across different settings/levels, to embed into practice, benefits of NAIT include links with autistic community – link with current research and links with ND pathway. You know if its coming from NAIT its been checked and is good to go, its using the right terminology and language, having consistency of approach across Scotland could be quite useful." [Deputy Head Teacher]*

**Context**	*"An unintended disconnect between grassroots and where things are strategically. The connection needs to be there and led…Work of NAIT needs to be fully understood strategically for it to have real meaning and impact between what's happening on the ground and at strategic level." [Third Sector Manager]*<br><br>*"I don't know if this is real or just a perception. With a move towards a Neurodevelopmental approach/pathways/services - has caused anxiety regarding increased workload, concerns that there will be a tsunami of work they cannot manage when workloads are already large. In [area] there has been a massive increase in requests for ADHD diagnosis, may be unintended impact of NAIT or whether it's the world we are in/other reasons. There is stress about how we will cope with the numbers, as we don't have systems in place, fear about increase in workload and insufficient resource." [Allied Health Professional, Autism Diagnosis]*<br><br>*"Dealing with change, so much change all at once and I think it's probably challenging for people…" [Autistic professional]*<br><br>*"Philosophical barrier professional socialisation issues – how do we influence this? Takes a lot to be different. Culture/ cross-sectoral working Early career professional development Understanding multi-disciplinary working." [Third Sector Manager, National]*<br><br>*"The NHS is overwhelmed." [Autistic Professional]*<br><br>*"Time and backfill to progress things when there is only one practitioner in the area to follow-up – barrier is facilities and resources in the area." [Medical staff, Autism Diagnosis]*<br><br>*"Aghast at different funding models across Scotland. They have 5 people for 4 hours per week. What can be achieved? They are taking time to set up the pathway, learn from others, learn from contacts at NAIT, try to be as efficient as possible, still so much they need to do, can only change incrementally." [Allied Health Professional]*
**Context**	*"More older people coming forward for autism assessment – [so] we need resources for older people." [Medical staff]*<br><br>*"With such limited resources we are only offering an assessment of autism service. Would like to offer sensory/AHP/environmental assessments, must be mindful of the resource. Works with [national autism group] for post diagnostic information booklet as a resource, as a clinician you feel so constrained and want them to have all to have assessment and environmental recommendations." [Allied Health Professional]*<br><br>*"Not that we don't offer but that we are not doing it consistently – such as parenting programmes are often funded short term, or criteria that only families known to our service can access, need a more joined up approach." [Clinical Lead Allied Health Professional]*<br><br>*"Philosophy of practice, how brave are we prepared to be? Need to be quite radical to come together, referral to meet at point of need, collective commitment, pace of change needs to speed up, change how we measure success*

	– not enough measures of things that as noted by [national review committee] , can we be held accountable? and not reductionist, engaging children and young people and families early/pre/post diagnosis." [Scottish Government Representative]<br><br>*"Policy is full of recommendations of what we should be –it's how we consistently translate that into real change" [Scottish Government Representative]*<br><br>*"Still work to be done to align all organisations in culture. Not down to individuals within organisations, NAIT promotes that but sometimes a disconnect between policy and what is happening." [Education Manager]*<br><br>*"It [change] has to happen across the country, because I don't think at the moment it does" [Autistic professional]*<br><br>*"…we need to have things integrated in a strategic way, need national leadership. It would be a waste if work is not maintained, finger on national and local leadership, acknowledgement that we need to do things differently so professionals have confidence, it comes back to ownership and leadership, we got a lot of good practice guidance – we need leadership for that to stay and grow." [Scottish Government Representative]*
