# Supplementary material for: An interdisciplinary nationwide complex intervention for lifespan neurodevelopmental service development: Underpinning principles and realist programme theory
Source: Front Rehabil Sci. 2023 Jan 30;3:1060596. doi: 10.3389/fresc.2022.1060596 (PMC9922833; doi:10.3389/fresc.2022.1060596)
Supplement: Supplementary file 2 [file Image1.pdf]

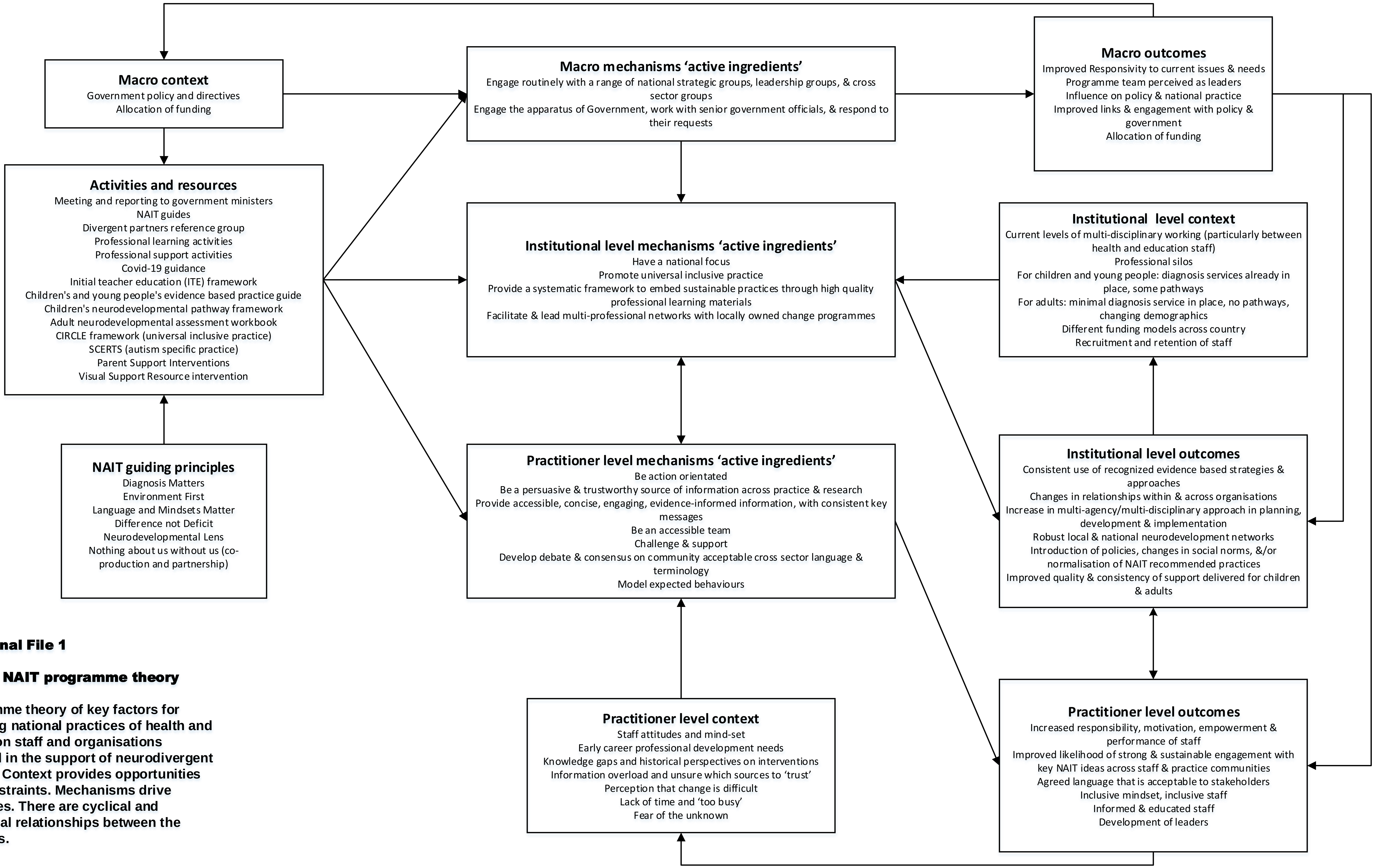

Additional File 1

Figure: NAIT programme theory

Programme theory of key factors for changing national practices of health and education staff and organisations involved in the support of neurodivergent people. Context provides opportunities and constraints. Mechanisms drive outcomes. There are cyclical and reciprocal relationships between the elements.
